# Textile Supplies for Hospitals

**Published:** 1916-07-08

**Authors:** 


### TEXTILE SUPPLIES FOR HOSPITALS.

### XIII.—The Care of Textiles.

Some difficulties in the care of textile supplies are to be minimised by the avoidance of large surplus stocks, in keeping the quantity in hand as nearly as may be to the quantity actively in use. The goods do not improve by keeping, albeit given certain precautions there is no reason why they should noticeably deteriorate. In the store cupboards of the linen-room the articles are little likely to encounter harm, although there may be more risk in leaving them in basements or outhouses. The goods require, of course, to be kept free from dust, as they readily may be in the papers or packages in which they arrive; and from damp. Granted that they are kept clean and dry it is even best to store them at atmospheric temperature, or at all events without considerable artificial heat. Darkness being favourable to the accumulation of dirt, is not desirable, although neither is direct sunlight. A cool, dry, shaded room, or one with a north aspect, is commonly regarded as best for the warehousing of textiles pending their entrance into use, and these are conditions which can often be met downstairs. Excessive moisture tends to set up mildew, and the starchy matters present in woven, unwashed cottons assist the formation of this fungoid growth. A mischief still more harmful is moth, and in some localities the penetration of moths which lay their eggs in cloth is difficult to exclude. They have a preference for undyed cloth, but they are unknown to attack unwashed blankets, in which the sulphur of the blanket-bleaching stove acts as a preventative. Few safeguards are more efficient than the herb wormwood hung in bunches from the roof or distributed in muslin bags, and experience shows this simple agent to be more dependable than the blocks of naphthalene which are often employed.

Upon the whole there is no more convenient form in which to buy piece-goods than in rolls wound around a flat board, although, if preferred, piece-goods can be delivered in flat folds, in what is technically known as the "plaited" or "cuttled" state. The roll is firmer and more compact, and the circumstances are uncommon in which the process of unrolling amounts to a trouble, as it may do in the case of long lengths of heavy goods. There is scarcely need to point out that a trim and handy form gives the best security against disorderliness and its concomitant waste. If tidiness does not secure heedfulness in use, at any rate untidiness definitely encourages disregard of the cost of material.

### Wear and Washing.

At best, the treatment to which articles of hospital wear are subjected is severe, and largely because of the frequency of laundering. The repeated wetting and drying with the incidental friction is weakening to their constitution, and the effects are accentuated when the drying is done at high temperatures. Under conditions which do not obtain in the laundry both wool and cotton are capable of disintegration by nothing more than water and heat. At 300° F. wet wool can be completely disorganised, so that it will rub to powder after being redried, and cotton begins to undergo disintegration at 350° F. These destructive conditions may be reproduced in sterilising textiles if not in washing them. Lower temperatures have little perceptible effect upon the cotton fibre, but materially lower ones act to the detriment of wool, and woollens should preferably be washed by hand at about blood heat.

The common experience of those who wash textile fibre in the course of its manufacture is that it pays handsomely to use the best-made and most neutral soaps. The extra outlay on soap is made to avoid a breakage of fibre which occurs if the material has been rendered brittle, and what applies in the instance of the loose unmanufactured fibre applies in a measure to finished goods. The enemy to be feared is free alkalinity, and all the more because strong alkali, when once absorbed, cannot all be washed out. Mere traces of caustic alkali present in cotton goods are enough in a certain length of time to rot their strength completely away in the parts affected. A strong lye dissolves the wool structure entirely, and a relatively weak one makes woollens harsh and robs them of their strength. It may very well happen that a small saving in soap is effected at a large expense in blankets and sheets and towels by shortening their lives. In point of fact there are textile manufacturers who save money by paying three times more for soap than there is any superficially apparent need to do. Notoriously, the public laundries in their zeal for a large turnover use materials which are not to the advantage of the clothes treated, but their interest and that of the owners are not entirely identical. It can be urged that one of the most hopeful ways of reducing the cost of maintenance in hospital linen lies in a careful supervision of the laundry. A beginning might be made with analyses of the soaps in use and an experiment with neutral soap at even materially higher prices need not be feared.

### Damage in Disinfection.

Where there is an option of using less or more destructive methods, the option ought to be exercised, as it can be in the laundry much more certainly than in the processes of disinfection. The sterilisation of soiled linen has not invariably been conducted with knowledge and discretion. Hospital porters have been to blame for rotting sheets by stoving them in sulphur fumes and converting them into something little better than tinder. Sulphur stoving is comparatively innocuous to woollens,

Note.—Previous articles in this series appeared on December 18, 1915, and January 8 and 22, February 5 and 19, March 4 and 18, April 1, 15, and 29, and May 13 and 27, 1916.

although all processes of disinfection are detrimental in their degree. Woollens are stained often and in part damaged in simple carbolising, and the indelibility of certain stains is probably to be explained by a chemical combination between the wool fibre and an acid. After impregnation the whole of an acid applied to wool can never be recovered by washing out.

Cottons and linens suffer less harm than wool from high degrees of dry or moist heat. Their strength is lowered temporarily whilst they are dehydrated, but in the main this is recovered when the fibre is free again to draw its natural content of water from the atmosphere. All the textile fibres are by nature hygroscopic and contain appreciable quantities of water even when to the touch they feel dry. The goods vary in moistness according to their surroundings, and are only reduced to a state of constant, or absolute, dryness by being heated in rising air currents for twenty or thirty minutes at 212 degrees F. or over. Conventionally dry cottons hold fractionally less than 8 per cent., by weight, of water, linens nearly 11 per cent., and woollens nearly 14 per cent., and they tend always to return to this or a greater state of moistness as they cool and draw humidity from the air. These are the normal percentages, and in containing such proportions the articles are in no sense damp to the touch. Without their natural quota of water the goods are lacking in suppleness, softness, and strength. Heat—and, beyond a certain range of temperature, moist heat—expels this water from the cells of the fibre, and this continual dehydration is set in motion both by the artificial drying of clothes in the laundry and by sterilisation in the steam oven. The process is not to the advantage of the goods, which are best dealt with at atmospheric temperature; but it cannot be suggested that the process is ordinarily avoidable in practice. The best that can be done is to avoid extremities and refrain from exposing valuable articles to treatments of more severity than is indispensably required by the needs of the case.

At the conclusion of this series it may be useful to offer in a compact form a short glossary of the technical terms to be met with in dealing with textile goods. The list is fairly exhaustive of those met with in general, although it does not profess to include terms of merely local application.

### Technical Terms.

Absorbent Wool.—Cotton deprived of its organic impurities.

Alhambras.—White figured quilts.

Alpaca.—Dress goods of Peruvian llama hair and cotton.

Ayrshire Blanket.—Also "Scotch" blanket. Woven with a twill and with little nap.

Batiste.—Fine white cotton, heavier than nainsook.

Botany.—Australian merino wool for the best costume serges.

Cloth Blanket.—Also "Bury" blanket. Woven plain, set close, and little napped.

Coutils.—Strong cottons with herringbone twill.

Damask.—Satin-faced linen in fancy designs.

Domette.—Imitation flannel, cotton, or wool and cotton.

Double Warp.—Made with two-fold warp yarn.

Drill.—Linen or cotton heavy cloth closely twilled.

Egyptian.—Fine cotton of brownish tint and Egyptian growth.

Fast Colour.—Fast to light, or to washing, rubbing, or perspiration.

Flannel.—All-wool plain fabric.

Flannelette.—Cotton cloth napped to give warmth and softness.

Galateas.—Strong striped cotton cloth.

Ginghams.—Plain cottons of checked pattern.

Glacé.—Glazed or polished, as in sewing-thread.

Grandrelle.—Shirtings with a warp of closely-twisted two-fold thread.

Greys.—Goods in the unbleached state, not necessarily true grey in colour.

Harvard.—Strong, closely-woven shirting.

Holland.—Brown or unbleached linen.

Huckaback.—Linen or cotton towels of firm structure and broken surface.

Indigo.—Dyed or printed with indigo, natural or artificial, wholly or in part.

Jaconette.—Cotton lawn.

Jacquards.—Patterned by means of the Jacquard loom.

Jobs.—Defective goods, sold at a reduction of price.

List.—The selvedge of cloth.

Long Cloths.—Plain or twilled bleached cottons for underclothing.

Madapollam.—Plain white cotton cloth of light weight.

Marseilles.—A thick cotton quilt.

Merino.—Merino hosiery is mixed wool and cotton. Merino wool is the fine wool of commerce.

Mohair.—Dress fabric of Angora goats' wool and cotton.

Nainsook.—Soft-finished plain cotton of light weight.

Natural.—Brown or grey coloured woollens of mixed shade, properly made by blending white and brown wool.

Oxford.—Shirtings softer than Harvards and usually in red or blue and white check.

Patent Quilt.—Smooth-surfaced cotton quilt with a raised figure.

Pick.—Otherwise "Shot." One thread of weft. "Picks per inch" denotes weft threads per inch.

Piece.—The unit of manufacture corresponding in length with that of the warp yarn; usually 50 to 70 yards.

Piqués.—Goods woven with parallel transverse ribs or cords.

Plain.—Plain weave is the regular one-and-one alternation of threads.

Prints.—Calico coloured in designs produced by printing, not by weaving.

Proofed.—Treated to resist rain.

Reed.—The number of warp threads in one inch.

Regattas.—Striped, strong cottons for uniforms.

Seconds.—Goods not of the first selection.

Serge.—Woollen or worsted, twilled.

Set.—The texture; cloths are said to be close or open set in accordance with the placing of the thread.

Shoddy.—The longer fibres recovered from breaking up used worsted cloth.

Size.—Glutinous matter added to assist weaving or to increase the weight of goods.

Ticking.—Containers for mattresses, strong, heavy linen or cotton.

Tow.—Surgeons' tow comes from jute. Short or matted fibre.

Truss.—A canvas-covered package.

Turkey-red.—Cotton dyed with alizarin oil.

Twill.—The diagonal rib produced in weaving.

Union.—Warp of one material, weft of another.

Warp.—The longitudinal threads of a fabric.

Waste.—By-products of any textile process.

Weft.—Transverse threads.

Witney Blanket.—Made in Witney, Oxfordshire.

Woollens.—Not synonymous with all-wool. Manufactured upon the woollen type of machinery, the fibres not arranged parallel.

Worsted.—Made of combed wool or of wools fairly uniform in length and parallel within the structure.

Zephyrs.—Cottons woven from coloured yarns.

6/4.—Six-quarter; 6×9=54 inches.

